# Developing public health surveillance dashboards: a scoping review on the design principles

**DOI:** 10.1186/s12889-024-17841-2

**Published:** 2024-02-06

**Authors:** Reza Rabiei, Peivand Bastani, Hossein Ahmadi, Shirin Dehghan, Sohrab Almasi

**Affiliations:** 1 Department of Health Information Technology and Management, School of Allied Medical Sciences, Shahid Beheshti University of Medical Sciences, Tehran, Iran; 2 College of Business, Government and Law, Flinders University, Adelaide, SA 5042 Australia; 3 Centre for Health Technology, Faculty of Health, University of Plymouth, Plymouth, PL4 8AA UK

**Keywords:** Public Health, Public Health Informatics, Public Health Surveillance, Dashboard

## Abstract

**Background:**

Public Health Dashboards (PHDs) facilitate the monitoring and prediction of disease outbreaks by continuously monitoring the health status of the community. This study aimed to identify design principles and determinants for developing public health surveillance dashboards.

**Methodology:**

This scoping review is based on Arksey and O'Malley's framework as included in JBI guidance. Four databases were used to review and present the proposed principles of designing PHDs: IEEE, PubMed, Web of Science, and Scopus. We considered articles published between January 1, 2010 and November 30, 2022. The final search of articles was done on November 30, 2022. Only articles in the English language were included. Qualitative synthesis and trend analysis were conducted.

**Results:**

Findings from sixty-seven articles out of 543 retrieved articles, which were eligible for analysis, indicate that most of the dashboards designed from 2020 onwards were at the national level for managing and monitoring COVID-19. Design principles for the public health dashboard were presented in five groups, i.e., considering aim and target users, appropriate content, interface, data analysis and presentation types, and infrastructure.

**Conclusion:**

Effective and efficient use of dashboards in public health surveillance requires implementing design principles to improve the functionality of these systems in monitoring and decision-making. Considering user requirements, developing a robust infrastructure for improving data accessibility, developing, and applying Key Performance Indicators (KPIs) for data processing and reporting purposes, and designing interactive and intuitive interfaces are key for successful design and development.

## Introduction

Public health surveillance is the continuous and systematic collection, analysis, and interpretation of health-related data essential for planning, implementing, and evaluating public health performance. It is a tool for estimating the health and behavior of a society, enabling the determination of health status and identification of interventions and their effects. Health monitoring empowers decision-makers for effective management based on valuable and up-to-date evidence [1]. Interpreting the obtained results and sharing the information helps the stakeholders take quick and appropriate measures to reduce morbidity and mortality and improve the welfare of society [2]. This process requires the cooperation of many stakeholders, from the community level to the senior management of the health system, who should work systematically and complementarily to promote public health security [3]. Public health goals include preventing epidemics, protecting against environmental hazards, encouraging and promoting healthy behaviors, managing natural disasters, assisting in community recovery, and ensuring quality and access to health services [4]. One of the essential services public health organizations provide is monitoring health status and identifying community health problems [4, 5].

### History of surveillance system and challenges

For this purpose, the public health monitoring system employs continuous monitoring systems to assess the health status of the community and utilizes the data for planning, implementation, and evaluation [4]. Initially, telephone reporting was used for public health monitoring. However, this method faced numerous challenges in analyzing and extracting valuable information for timely decision-making due to the production of large and complex data sets.

Evidence shows that the generation of substantial data amounts led to information overload at high organizational levels. Consequently, these data sets were rarely utilized for decision-making in practice in an effective way. The process of reporting, collecting, and analyzing the data often extended over several weeks, impeding a targeted and timely response [5, 6].

With the advent and popularization of the Internet, a suitable platform was provided for the swift collection of society health-related data from a wide range of available electronic data sources. The first initiative of this kind was the Program for Monitoring Emerging Diseases (ProMED-Mail), launched in 1994 as the communication system of the Program for Monitoring Emerging Diseases. Subsequently, the World Health Organization (WHO) established an effectively organized infrastructure called the Global Outbreak Alert Response Network (GOARN) [7].

Today, public health monitoring systems can swiftly collect necessary data from different parts of society, including remote areas, to obtain essential information for identifying early events and preparing for them [7, 8]. Studies demonstrate that, despite the clear advantages these systems offer compared to traditional surveillance systems, they still face unresolved limitations. The key limitation of other surveillance systems, in contrast to dashboards, is their inability to analyze and extract valuable information for timely decision-making and the lack of integration and collection of information from different sources. Given the large volume of data and the unstructured nature of data sources, methods are required to extract, process, and analyze the data, presenting the interpreted information most effectively to users [9].

### Dashboard in public health surveillance

Considering the extensive data sources and the diversity of potential users in public health monitoring systems, dashboards can serve as a suitable tool to facilitate production and provide information to managers and policymakers in this field. In recent years, with the increase in the global spread of infectious diseases that have the potential to become epidemics and pandemics [10, 11], the importance of utilizing Public Health Dashboards (PHDs) in continuously monitoring the health status of communities, timely diagnosis, and proper management of these diseases has significantly increased. The advent of the COVID-19 pandemic further emphasized the importance of using real-time data to manage and control this disease at the societal level, making the role of PHDs more prominent [12, 13].

Dashboards serve as decision support tools, presenting essential business information in graphic and visual form. They can retrieve and analyze a large amount of data by interacting with various data sources, extracting information from databases, and delivering results based on Key Performance Indicators (KPIs). As a result, dashboard users can quickly gain insights into the current situation and progress of the business. When designing dashboards, it is necessary to choose KPIs that align with users' needs. Appropriate KPIs should be selected and organized based on the dashboard`s objectives and its users. The effectiveness of KPIs is maximized when the dashboard displays indicators that resonate with users' understanding and knowledge. Furthermore, the careful consideration of the number of selected KPIs for monitoring by the dashboard is essential [14–16].

The PHDs aim to facilitate the continuous monitoring of the health status of the community and the monitoring and prediction of disease outbreaks by collecting and integrating real-time data from various data sources. They assist in managing and controlling diseases by displaying KPIs in a well-designed user interface [17]. Therefore, considering the volume of data and the need for real-time monitoring and response in public health situations, attention to dashboard design principles for public health surveillance is essential [18].

Studies on PHDs principles primarily focus the content and user interface of these systems. The suggested design principles in these studies include a customizable, actionable "launch pad" [19, 20], supporting correct data interpretation [20, 21], information aggregation [22], minimalist aesthetics [21], user workload reduction [21], GIS interface [23], minimal cognitive processing, and the use of temporal trend analysis techniques [24]. In other words, the design principles suggested in the studies primarily focus on the content and user interface of these systems. Additionally, our study's results section highlights other features that should be considered in the design of public health dashboards.

This study was conducted to identify the design principles of PHDs not only focusing on the content and user interface aspects but also presenting a comprehensive view of all key design principles of PHDs. The aim is to provide insight for public health policymakers to facilitate and accelerate decision-making in epidemics and medical crises by extracting data from various systems and sources and providing timely reports.

## Methodology

### Study design

Scoping reviews try to identify, retrieve, and summarize information from studies relevant to a particular topic to find key concepts. They are conducted to map the body of the literature on a topic area [25]. One of their advantages is determining the feasibility and necessity of conducting a systematic review in a specific domain [25]. Available knowledge indicates that the research question could be considered a dominant factor in designing a scoping review or a systematic one. With a research question addressing the feasibility, appropriateness, meaningfulness or effectiveness of a specific treatment or practice, systematic review is preferred. In contrast, when the authors aim to identify specific characteristics/concepts in the studies, mapping, reporting or discussing these characteristics/concepts, a scoping review is preferred [26].

Based on the present RQ, Arksey and O’Malley’s framework (2005), as an influential framework suggested by the JBI guidance, was applied to conduct this scoping review [25]. Six following stages are recommended based on this framework; the first five are compulsory for the robustness and trustworthiness of the review, while the last stage is indicated as an optional one.

### Identifying the research question

The question should incorporate the population (or participants) /concept /context (PCC) elements per the guideline. This study included all the published papers about PHDs. The context refers to all the principles and determinants that impact designing such dashboards, and it also refers to applying PHDs in decision-making and monitoring the health status. Accordingly, the main research question is: “What are the key design principles of a public health dashboard?”.

### Identification of relevant studies

Searches were conducted in PubMed, Web of Science, IEEE, and Scopus. A combination of MeSH terms and related keywords was used for the search strategy. The search strategy was carried out with the following keywords.

(("Surveillance"[Title/Abstract] OR "Public Health Surveillance"[Mesh] OR "public health"[Mesh] OR "public health"[Title/Abstract]) AND) dashboard [Title/Abstract] OR “Web-based surveillance system” [Title/Abstract])(

The search was carried out for articles published between January 1, 2010 and November 30, 2022. The final search of articles was conducted on November 30, 2022. EndNote version 20.2.1 was applied to manage the articles` inclusion and screening process.

### Study selection

For this purpose, first, the retrieved articles were screened based on their title and abstract. Two authors reviewed all these titles and abstracts independently, and the senior author (RR) finalized the cases of disagreement. After the approval of the remaining articles by the senior author, the articles` full text was independently reviewed by two authors based on the inclusion and exclusion criteria of the study (Table 1). Any disagreement regarding the selection of articles was discussed with the senior author. Preferred Reporting Items for Systematic Reviews and Meta-Analyzes Extension for Scoping Review (PRISMA-ScR) guideline [27] was used to manage the eligible articles at this stage.

**Table 1:** Eligibility criteria

**Inclusion criteria**
• Studies focused on PHDs were used for geographic monitoring and tracking of public health or disease surveillance
• Studies that focused on the development and implementation of dashboards at the global, national, provincial or local levels
• Studies on web-based surveillance systems equipped with a dashboard
**Exclusion criteria**
• Peer-reviewed articles focused on the development, implementation, and/or evaluation of a dashboard used in healthcare settings, including clinics, hospitals, health systems, or any other settings where medical care is provided. (Based on the aim of this study to review the PHDs, we exclude those dashboards targeted at medical care centers due to the different nature, capabilities and features of the public health dashboard are those dashboards used in medical care centers)
• Non–English publications
• Studies published before 2000

### Charting data

The descriptive data extracted from the articles, including the year of publication, public health category, study setting, and dashboard implementation level, was inserted into Microsoft Excel Version 16 (Microsoft Corporation, Redmont, WA) for combination and analysis.

In this step, two data analysis methods, quantitative descriptive analysis and qualitative content analysis, were applied. Excel software (version 16) was used to summarize the distribution and frequency of the included articles based on year of publication, public health category, setting of the study, place of conducting the study, and dashboard implementation level (level of implementation of the dashboard at the global, national, or local levels). Then, the design principles of the PHDs were extracted by reviewing the content of the articles (Table 2).

**Table 2:** Principles for designing public health dashboard

Main principle	Subsidiary principles		Reference
Considering aim and target users	✓ Knowing the audience and their information needs ✓ Level (scale) of focus ✓ Responsible organization and type ✓ Multilanguages available ✓ Scope of web page information ✓ State the purpose of dashboard		[28–30]
Appropriate Content	KPI	✓ Macro, Mezzo, Micro level KPIs ✓ Timely and actionable indicators based on health system capacity ✓ Including relevant data disaggregation options (Sex, SES) **✓ Managing the type, volume, and flow of displayed information** **-Disaggregating the information into relevant subgroups**	[28, 29, 31–41]
Interface	Interaction techniques	✓ Provide overview of KPIs, change the display size and location information ✓ Zoom in and zoom out, pop-up and control commands and warning, customizable and actionable dashboard ✓ Switching from a global to local view, drill down to the local regions of the map to explore datasets in greater detail, not used of scrolling	[4, 28, 29, 34, 36–38, 40, 42–49]
	Visualization techniques	✓ Choosing the right data visualization ✓ Visualization techniques of data tables, pie charts, bar, histogram, line, area, scatter, bubble and a series of multiple and interactive maps, equipped with geographic information system (GIS) software ✓ Using storytelling and visual cues ✓ Supporting Correct Data Interpretation (using colored markers for clients to indicate their status, highlighting urgent/emergency alerts in red, and showing the data lines in the charts as blue (routine and exercise alerts) or red (urgent and emergency alerts) ✓ Minimizing distractions, clichés, and unnecessary embellishments (routine and exercise alerts) or red (urgent and emergency alerts)	[28, 29, 34, 35, 37, 42, 44, 47, 50–59]
Considering types of data analysis and presentation	Trend Analysis, tracking, and forecasting	✓ Provide real-time analysis ✓ Linking time trends to policy decisions ✓ Geographic levels of analysis ✓ Global and local comparison ✓ Chart selection, mini map, and global information display ✓ Techniques to analyze time trends and viewing past data ✓ Show trends and changes in data over time ✓ Key numbers relating to a region ✓ Assessing performance ✓ Support identification and evaluation of trends over time	[29, 34, 36, 38, 45, 53, 55, 60–62]
	Applies machine intelligence	✓ Anticipate spread and assess patterns ✓ Allow users to select the time period over which performance indicators are displayed ✓ Support comparison against the national average	[53, 55, 60, 61]
	Reporting format	✓ Reports in Word and PDF	[29, 34, 36, 38, 45, 54, 56, 61–63]
Infrastructure	Data integration and warehousing	✓ Proper design of data warehouse and data collection ✓ Data integration with online analytical processing system and data warehouse or other systems ✓ Data warehouse integrated with process data and operational security and data close to real-time ✓ The architecture based on the service-oriented architecture (SOA)	[45, 46, 51, 63]
	Integration data Sources and data generation	✓ Reporting data sources and methods clearly for trust to the dashboard ✓ Data quality was assessed by examining accuracy, real-time, and completeness ✓ Data Input, Storage, and Extraction process for the extraction of data warehousing ✓ Providing reliable, accurate, consistent and timely data	[29, 31, 32, 34, 42–44, 48, 50, 59, 64, 65]
	Data quality	✓ Completeness (e.g., missing data), correctness (e.g., accuracy), currency (e.g., timeliness), and provenance (e.g., reliability of the source)	[28, 30, 37, 40]
	Information standards	✓ Information Exchange standards and content standards ✓ Privacy and security standards, Functional standards (Work processes, workflow and dataflow models) ✓ Standard inputs for the dashboard frontend ✓ Standard architecture for including new datasets into the dashboard ✓ Standard dataset formats for the generation of data visualizations ✓ Data collection, data fusion logic, data curation and sharing, anomaly detection, data corrections, and the supportive human resources	[28, 30, 35, 37, 40, 42, 52, 55, 56, 59]
	System security	✓ Methods, techniques, and technologies used to protect data security, attention to system security	[28, 30, 40]
	Accessibility	✓ Web and mobile access, desktops, laptops, and tablets	[55, 56, 59]

For qualitative thematic analysis, the findings of the studies were examined line by line, and the primary codes were extracted for formulating the research question. After extracting the initial codes and reviewing these, the final codes were emerged and subsequently categorized to create subsidiary principles that ultimately led to a higher conceptual level.

Microsoft Packages Office 360 was used to categorize the design principles of dashboards. This scoping review also utilized trend analysis to illustrate the trends of publications in each of the public health categories. The number of articles published in different years was drawn using Microsoft Excel (Version 16).

## Results

**Fig. 1:**
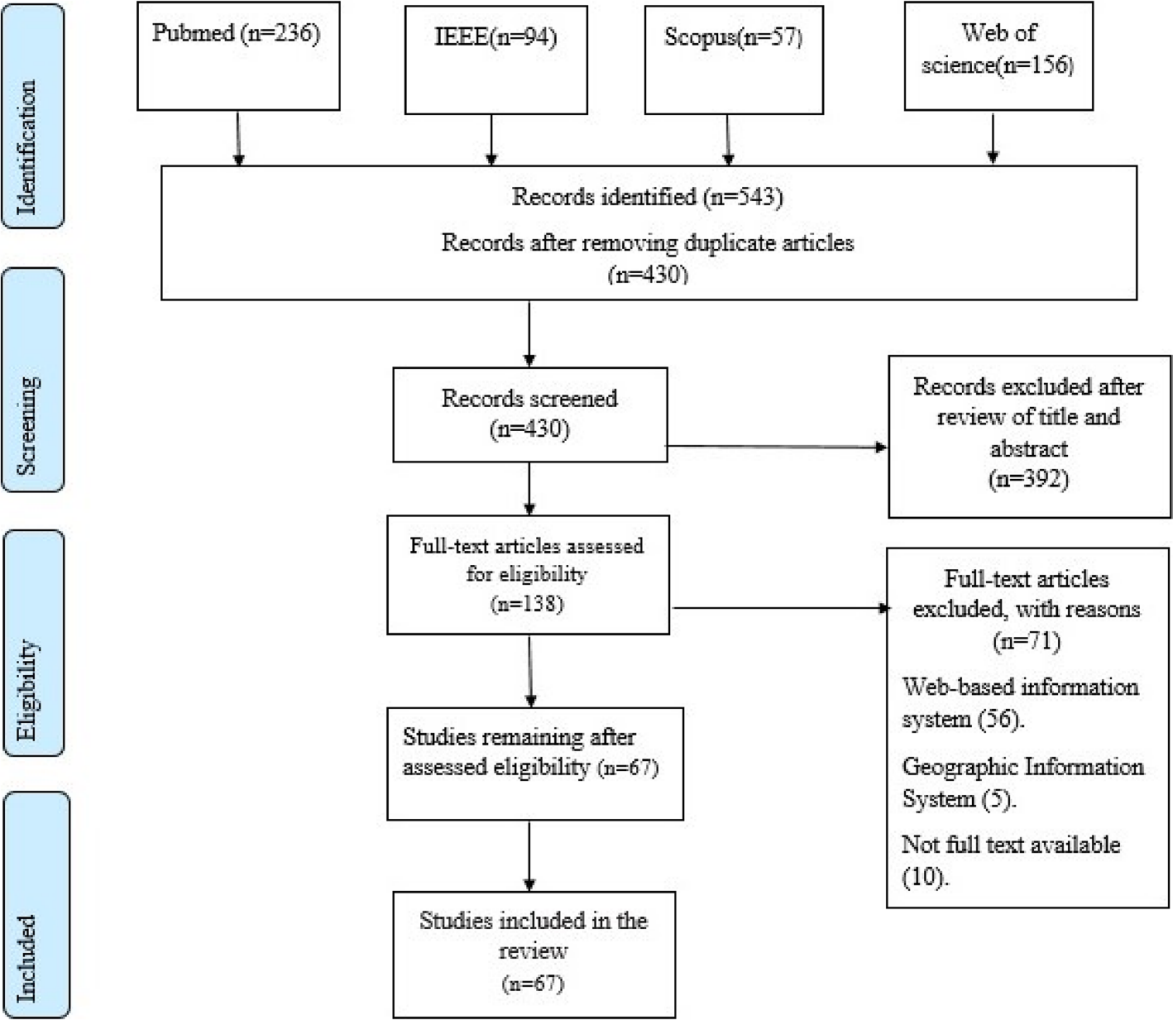
Flow diagram of conducting searches, filtering and paper selection

A total of 543 articles were retrieved after searching the databases. The PRISMA flow diagram illustrates that 67 articles were eligible for analysis based on the inclusion and exclusion criteria after eliminating the duplications and screening the articles. (Fig. 1).

### Characteristics of included studies

**Fig. 2:**
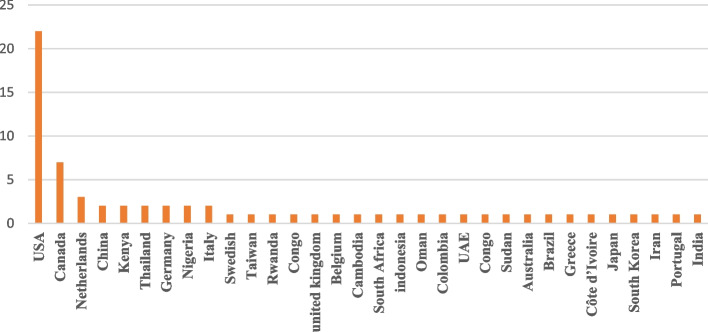
Geographical distribution of studies

**Fig. 3:**
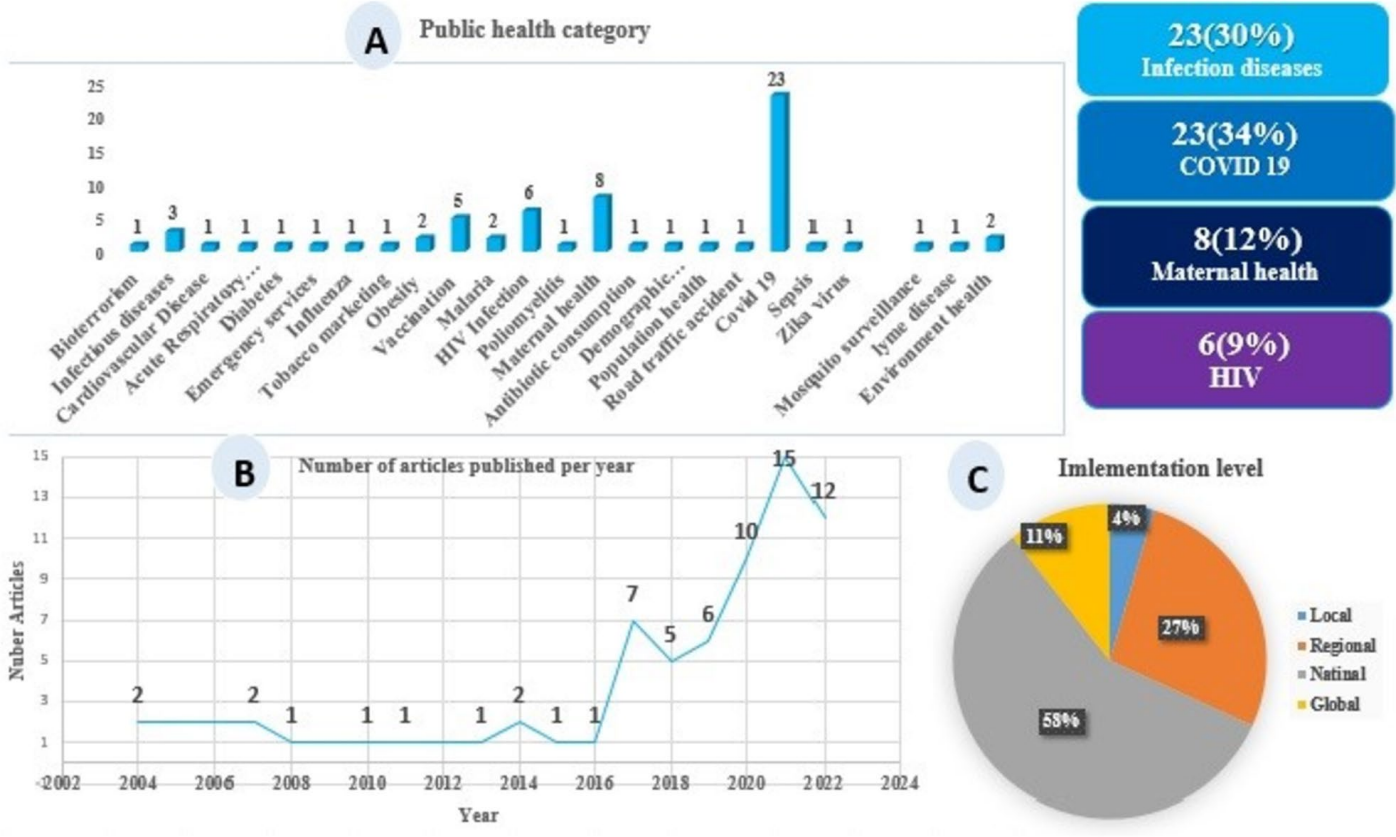
**A**) Public health category, **B**) Number of articles published per year, **C**) Level implementation of PHDs

The geographical distribution of the designed dashboards showed that most of the selected studies were conducted in North America (*N* = 29, 43%), Europe, Asia, and Africa, respectively (Fig. 2). About the studies conducted on PHDs, there was an increasing trend in the number of published articles from 2020 to 2022. Regarding implementation scale, the designed dashboards were mainly reported at the national level (58%) (Regional 27%, local 11%, and global 4%). In addition, (*N* = 23, 30%) of dashboards were designed to monitor and control for COVID-19; followed by dashboards developed for maternal and newborn health (*N* = 8, 12%) and AIDS (*N* = 6, 9%) (Fig. 3).

### Principles of designing PHDs

#### Considering the objective and target users

First, the purpose of designing a dashboard and the target users should be considered. The dashboard's design, visualization tools, content, and how the information is represented vary based on the dashboard users. In the study of Véronique et al. in the Netherlands, to investigate the development and actionability of the COVID-19 dashboard, it is important to specify the purpose and users of the dashboard in designing the dashboard [28]. In a review of 158 dashboards from 53 countries, Ivankovi et al. identified seven common features among them. “Know their audience and information needs” is mentioned as the first feature in the principles of designing PHDs [29]. Therefore, the need for compatibility between the content and information displayed by the dashboard and the tasks and needs of users can impact the use of the dashboard [28–30].

### Appropriate content

Véronique et al. [28), introduced content and data, and Ivanković et al. [29], presented managing the type, volume, and flow of displayed information, as public health dashboard design features.

In the reviewed dashboards, KPIs were placed on the dashboard's main page, allowing for timely monitoring and display of the current situation at a glance. KPIs' placement and display in the dashboard is top-down so that macro indicators (global, national) (for example, number of deaths due to COVID-19 global or by county) are placed on the main screen. KPIs and global indicators can be compared at this level. Mezzo (urban, regional) (for example number of deaths due to covid-19 at global or by region or cities) indicators are at the next level, which can compare cities and regions. Micro indicators (for example, the number of deaths due to COVID-19 at hospitals) are on the third level, which are performance indicators at the level of institutions. Managing the amount of information displayed on the dashboard is also essential [28, 29, 31–41].

### Interface

The dashboard user interface consists of two parts: interactive tools and visual tools.

#### Interactive tools

In the reviewed dashboards, the summary view feature was first used to monitor macro indicators at a glance, and unnecessary details were not displayed. This feature helps summarize data and reduce complexity. The indicators' details can be accessed using the drill-up and drill-down features if needed. The pan-and-zoom feature can be used to magnify or reduce the details. The customizable feature enables users to customize information display based on indicators according to their needs. If real-time monitoring is needed, the reports based on the determined KPIs are displayed in real-time [4, 28, 31, 34, 36–38, 40, 42–49].

#### Visual tools

Using appropriate visualization techniques based on KPIs' nature and users' experience and skill will improve dashboard design. Choosing the correct type of visualization tool that matches the type and nature of KPIs is essential in designing dashboards. In the reviewed dashboards, different visual techniques (including data tables, pie charts, bars, histograms, lines, areas, scatter bubbles, and a series of multiple and interactive maps) were used based on the nature of the indicators [29, 34, 35, 37]. In the study of Ivanković et al., by examining 158 PHDs, various types of visualization to display information in the dashboard include time trend analysis availability, use of time trend analysis, geographic levels (scales) of analysis, disaggregation options, use of narratives to interpret data [29].

In Lee et al. study, visual summarizations (e.g., heat map and time series chart) and interactive tools (e.g., year selection, automatic year play, map zoom, copy or print data, ranking data by name or value, and data search) were implemented to enhance user experience [37]. Correspondingly, data interpretation tools (For example, using color coding to indicate urgent/emergency alerts with red, normal situations with green, and warnings with yellow; minimizing distractions; avoiding unnecessary visual decorations in dashboard design) are essential in dashboard design [28, 29, 34, 35, 37, 42, 44, 47, 50–59].

### Considering the types of data analysis and presentation

Data analysis helps users understand the relationships between data and trends in the dashboard [29, 34]. Various types of analysis were in the reviewed dashboards, including analysis at different geographic levels, comparing global and local KPIs, comparing indicators with standard values, and presenting data or reports in the format required by the users, such as Word or PDF [29, 34, 36, 38, 45, 53, 55, 60–62]. In the study by Cheng et al., the features for efficient data presentation are suggested: (1) provision of information that viewers need quickly and clearly, (2) organization of information to support meaning and usability, (3) minimization of distractions, clichés, and unnecessary embellishments that could create confusion, (4) creation of an aesthetically pleasing viewing experience, and (5) consistency of design for easy data comparison [45]. Artificial intelligence and data mining techniques can be used to predict trends and patterns in data over time [53, 55, 60–62].

### Infrastructure

The infrastructure and implementation of the data warehouse are vital in designing dashboards and facilitating the collection and management of data from different sources. Data warehouses are central repositories of integrated data from one or more disparate sources. A dashboard pulls the data from your data warehouse and transforms it into a series of charts, graphs and other visualizations that update in real-time. The data warehouse is used to collect and manage data from various sources, and it can be used for reporting, reviewing, and analyzing data if equipped with a dashboard [45, 46, 51, 63]. High-quality data is essential for an effective data warehouse. It is crucial to have a standard for data transfer and check the data quality before storing it in the data warehouse. Data quality aspects in the examined dashboards included data completeness (e.g., missing data), correctness (e.g., accuracy), currency (e.g., timeliness), and provenance (e.g., reliability of the source). The standards included content, transmission, structural, and security [28, 30, 35, 37, 40, 42, 52, 55, 56, 59]. Transferring data between systems and creating interactions between data sources requires attention to security and data access. For security measures, all users are assigned a level based on their performance and duties in the authorization system. Three levels of data security were implemented in the reviewed dashboards, i.e., client level, data transfer level, and server level. At the client level, user authentication is checked every 10 min to prevent cyber-attacks and interference in database queries through SQL injection [40, 42, 52]. The client and server data were encrypted through NoSSL open-source software at the data transfer level [52, 55].

Given that the web server is open to public access, a backup computer in the middle (intermediary computer) is needed for filtering access to the database [28, 30, 40] to ensure proper security standards and protect the central database. This means all requests are passed through the web server to the intermediary computer, then to the central database, and vice versa. The dashboard design should consider easy access to the dashboard via phone, tablet, and laptop for real-time monitoring and checking KPIs at a glance [55, 56, 59].

## Discussion

### Main findings

This scoping review study aimed to determine the design principles of PHDs. The included articles explained the details of the design and development of PHDs and their design criteria. The study findings revealed that the production rate of PHDs has been increasing in the past few years. The emergence of COVID-19 and the efforts to manage and control the outbreak/pandemic have significantly impacted this increasing trend. Several institutions worldwide have designed and developed COVID-19 dashboards to report epidemiologic statistics on a county, state, or national scale. Almost all states and most major cities in the USA had deployed a COVID-19 dashboard by the end of 2020. By 2021, all dashboards designed for this purpose had been updated to include information on vaccination or separate dashboards had been created to track COVID-19 vaccination [13]. Due to the massive amount of data and the need for real-time monitoring and response in public health situations, it is essential to pay attention to dashboard design principles to support the goals of public health surveillance [18]. After examining the indicators presented in the reviewed studies, dashboard design objectives and target users, dashboard content, dashboard user interface, data analysis and display, and infrastructure were identified as five general and essential principles in designing PHDs. Studies have also discussed the requirements and design principles of PHDs. Identifying users and their needs, using narrative information in addition to quantitative information in the dashboard, using a geographic map to display location data better, and stating the source of the data reported by the dashboard are mentioned criteria for designing a dashboard [66].

Likewise, the necessary components to support and facilitate implementing dashboards in public health organizations have been mentioned, including storage and management of data and information from different sources, coordination of data from different sources, standards support, analysis, defining and identifying KPIs, and information visualization [13]. Rasmussen et al. suggested four general principles for designing dashboards: presentation format, integration, interface design, and development and implementation [67]. These researchers remarked that inadequate attention to these principles could result in challenges for PHDs [67]. Furthermore, Ghazi Saeedi et al. mentioned KPI development, data sources, data generation, integration of dashboards to source systems, and information presentation issues as the challenges of implementing PHDs [68].

### Purpose and users

The purpose of designing a dashboard is to provide a suitable tool for exploring a data set and finding the information the user needs. Therefore, paying attention to the user's needs and designing the appropriate dashboard is particularly important. Considering that a variety of users use dashboards, it is impossible to design a dashboard that fits the personality and ability of each user. However, identifying the primary goal of designing a dashboard and its target user group is the first step in choosing the correct and accurate KPIs, defining appropriate interactive and visual tools, and considering related data analysis methods. Marshal et al. have also emphasized the importance of this principle in designing PHDs in two separate studies [69].

### Content

KPIs are the main content component of a health dashboard. Therefore, choosing the type and number of indicators the dashboard should monitor and display is essential in designing and developing dashboards [32, 70, 71]. Every organization must measure the indicators that fit its objectives [72]. After identifying the main objective and target users, it is necessary to determine the appropriate measurement indicators. Determining a specific and adequate number of indicators emphasizes the available information, and users can review all the indicators at a glance. These findings are consistent with Peters et al.'s study, which indicated that moderate use of indicators can display information in various ways and effectively guide the user's visual flow by creating a particular order [73, 74]. Serb et al. also suggested the importance of organizing indicators in the dashboard according to the level of use (macro, mezzo, micro level). Their study showed that at least 15 to 25 indicators are required for monitoring purposes in dashboards [75].

### Interface

In user interface design, attention to the principles of information visualization and interaction with the user interface is essential [76, 77]. Uniform techniques were not used to visualize functional indicators in the reviewed studies. Uniform visualization techniques are ineffective in dashboard design since it is necessary to consider users' preferences, abilities, knowledge, and skills in visualizing dashboards. Besides, Steichen and Mawad pointed out in separate studies that creating adaptive and personalized visualization systems tailored to users' cognitive and individual abilities can lead to a better understanding of displayed information [78]. The nature of data and human factors such as experience, skill, cognitive styles, and user preferences are also influential in selecting visualization and interactive techniques [79, 80]. In Shneiderman's study, interactive techniques included "overview, zoom, filter, details-on-demand, relate, history, and extract" [81]. Khan et al. indicated that interactive techniques included "zoom and pan, overview and detail, and filtering" [82]. In Dal et al. 's study, interactive techniques for the dashboard included controlling the level of detail, filtering, searching, and customizing the display [83]. Yi et al. similarly implied interactive features included "select, explore, reconfigure, encode, abstract/elaborate, filter, and connect" [76].

### Types of analysis and data presentation

The main application of dashboards is data analysis to provide appropriate insights into the regional distribution of disease burden and help allocate resources correctly. This analysis can help policymakers and healthcare providers make appropriate decisions. In most studies, timely data reporting and a suitable time trend in data analysis have been proposed as essential indicators in dashboard design. These findings align with the results of Curriero et al., emphasizing the importance of providing up-to-date data reports [57]. Another critical indicator in dashboard design is the ability to analyze data based on geographic location, age, gender, social status, ethnicity, and race. By collecting, registering, and using data related to meaningful subgroups of the population, these critical (and changeable) differences might be noticed. Brehaut et al. also showed that as far as infrastructure limitations and legal barriers allow, these indicators are vital and should be considered in designing a dashboard. Finally, some studies used descriptive approaches, machine learning prediction models, and simulations to predict future situations [84]. This indicator can help control diseases, especially pandemics [85]. This issue was also raised as one of the indicators that can help increase the efficiency of these dashboards in Brehaut's research [84].

### Infrastructure

Infrastructure is the backbone of every system, and the successful adoption of any eHealth system depends on the infrastructural arrangements [86].

The findings of this study revealed that a high percentage of studies had mentioned data warehousing and appropriate web service architecture as necessary infrastructures for dashboard design [67, 87]. Given the diversity of systems and data in different formats, the dashboard infrastructure's main challenge is data integration, and creating data warehouses is an appropriate solution to this challenge [88, 89]. Access to appropriate software and hardware, use of modern technology, sharing reliable and up-to-date data, and the need for a capable workforce to create and maintain dashboards are other identified components related to dashboard infrastructure [90].

In addition, the necessary infrastructure for creating a dashboard includes access to modern IT software and hardware, continuous and reliable data sharing, and the need for a capable workforce to create and maintain dashboards [13]. Among the challenges associated with PHDs are data quality, big data, information architecture, privacy, and security [91]. The quality of stored data is also one of the critical issues in dashboard infrastructure. Given the importance of data in decision-making at the public health level, the quality of stored data is also an essential prerequisite for dashboard infrastructure. Fadahunsi et al. also considered data quality an essential dashboard infrastructure component in two separate studies [92].

Informativeness (accuracy, completeness, interpretability, plausibility, provenance, and relevance), availability (accessibility, portability, security, and timeliness), and usability (conformance, consistency, and maintainability) are key features indicated in these two studies [92, 93]. Transparency about data sources and how indicators are calculated are critical for reports' overall quality, credibility, and reliability. Identifying the sources used and calculating indicators in PHDs are essential for transparency about data collection and would help to understand the logic behind the reports [73, 94].

Regarding infrastructure, information security was also one of the issues mentioned in a considerable number of sources. Given the integration of various systems at the organizational level and their connection to the dashboard, using data exchange standards for system interaction is an issue that should be considered [95]. These findings were in line with a study by Li Y-CJ et al., who considered electronic data exchange in standard data formats essential for improving data accessibility [96]. Moreover, this study showed that these standards preserve data security, reduce resource waste, and improve the quality of care [96]. Based on the importance and quality of the disclosed information, access control should exist at multiple levels of security/privacy [97].

### Implications for policy, practice, and future research

This study extracts the public health dashboard's design criteria and proposes some design principles based on the available knowledge in the area. Given the enormous volume of data and the need for quick response in public health situations, this study is a potentially vital source for helping policymakers, developers, public healthcare organizations, and managers to design and develop PHDs as a prerequisite for early response, particularly during the probable pandemic. As pandemic response requires early and robust verifications, identifying this potentiality of dashboards in data management can be helpful. The lesson learned from the COVID-19 pandemic indicates that public health organizations must equip themselves with dashboards for emerging pandemics and many other vital activities for public health promotion. In other words, investing in dashboard software tools and systems, processes, and people who support PHDs, could be a tailored practice and intervention for the public health policymakers. Exchanging information between healthcare providers and public health organizations and developing an appropriate infrastructure for data exchange is critical for more effective monitoring of epidemic diseases. Clinical information systems should exchange information in real-time at a national level to effectively use dashboards at the public health level for monitoring and managing epidemic diseases and taking timely actions. Therefore, it is suggested that the government examines the technical infrastructure (data architectures, structural and content standards, data exchange, security, and data resources) for appropriate data exchange between various clinical systems and the dashboard.

### Strengths and limitations

The present study addresses the principles of designing PHDs and provides a comprehensive view of designing dashboards. In addition, this study investigated all aspects of PHDs design, including purposes, content, user interface, types of analysis, and infrastructure, and proposed sub-criteria for each criterion. However, the study needed further access to some articles' full text and the search was also restricted to articles published in English.

Although the scoping reviews are mainly designed to help policymakers figure out the key concepts underpinning a research area and help them to have clear working definitions, and/or the conceptual boundaries of a topic, the results of this study need to be customized and tailored based on the local public health priorities of the countries through Focus Group Discussions (FGDs) and feasibility assessment panels before applying at the implementation phases. It is also suggested to conduct a study regarding the design and implementation of PHDs according to the income level of the countries. The results of this scoping review can open a new window for conducting future systematic reviews to address the feasibility, appropriateness, meaningfulness, or effectiveness of public health surveillance dashboards. Finally, as the descriptive results present a geographical distribution of PHDs implementation to create a general understanding and illustrate a map to policymakers, stakeholders and researchers to figure out the concentration hotspots and healthcare system`s attention to the topic, it is important to interpret the results conservatively to avoid any kind of misinterpretation about the place or type of the included studies. The same limitation could be considered as the present results were not broken down by country (low, middle and high income), so the findings should be generalized conservatively to the setting of low-income countries as most of the included studies were conducted in high income countries.

## Conclusion

Monitoring health, managing epidemics, and taking timely action requires real-time information exchange between clinical information systems and PHDs. Therefore, given the volume of data, the need for real-time monitoring and response in public health situations, and disease surveillance during epidemics, it is necessary to pay attention to dashboard design principles to achieve public health surveillance goals. Findings of the current indicated that design principles for the PHDs could be presented in five groups, i.e., considering aim and target users, appropriate content, interface, data analysis and presentation types, and infrastructure.

## Data Availability

All data generated or analyzed during this study are included in this published article.

## Abbreviations

PHDs: Public Health Dashboards
ProMED: Program for Monitoring Emerging Diseases
GOARN: Global Outbreak Alert Response Network
PCC: Population (or participants)/Concept/Context
GIS: Geographic Information System
KPI: Key Performance Indicators
AIDS: Acquired Immunodeficiency Syndrome
SOA: Service-Oriented Architecture
FGDs: Focus Group Discussions
